# Effect of low frequency sound vibration on acute stress response in university students—Pilot randomized controlled trial

**DOI:** 10.3389/fpsyg.2022.980756

**Published:** 2022-10-13

**Authors:** Jiří Kantor, Zdeněk Vilímek, Martin Vítězník, Pavel Smrčka, Elsa A. Campbell, Monika Bucharová, Jana Grohmannová, Gabriela Špinarová, Kateřina Janíčková, Jian Du, Jiaoli Li, Markéta Janátová, Vojtěch Regec, Kristýna Krahulcová, Lucia Kantorová

**Affiliations:** ^1^Center of Evidence-Based Education and Arts Therapies: A JBI Affiliated Group, Faculty of Education, Palacký University Olomouc, Olomouc, Czechia; ^2^Faculty of Education, Institute of Special Education Studies, Palacký University Olomouc, Olomouc, Czechia; ^3^Department of Information and Communication Technologies in Medicine, Faculty of Biomedical Engineering, Czech Technical University in Prague, Kladno, Czechia; ^4^VIBRAC Skille-Lehikoinen Centre for Vibroacoustic Therapy and Research, Eino Roiha Institute, Jyväskylä, Finland; ^5^The Czech National Centre for Evidence-Based Healthcare and Knowledge Translation (Cochrane Czech Republic, Czech CEBHC: JBI Centre of Excellence, Masaryk University GRADE Centre), Faculty of Medicine, Institute of Biostatistics and Analyses, Masaryk University, Brno, Czechia

**Keywords:** heart rate variability, university, Vibrobed, music, vibroacoustic therapy, stress

## Abstract

**Background:**

Low frequency sound (LFS, combined with music listening) is applied by practitioners in vibroacoustic therapy who report a positive effect of this intervention on acute stress response. However, there is a lack of research on this topic and studies with mainly objective measurements are scarce.

**Materials and methods:**

In this pilot double-blinded Randomized Controlled Trial we used a multimodal approach to measurement of acute stress response in 54 international university students attending a university summer school in Olomouc, the Czech Republic who were individually randomized into a group receiving LFS vibration and a control group. In both groups, the acute stress response was measured by heart rate variability (HRV), visual analogue scales (VAS) for stress and muscle relaxation.

**Results:**

Differences were found in pre-test post-test measures, however, between groups differences occurred only for HRV, with statistically significant improvement in the experimental group (parameter LF/HF and pNN50).

**Conclusion:**

Vibroacoustic therapy has the potential to contribute to the stress management of university students. Further research is needed to explore the effect of LFS on stress response, especially when applied without additional music listening.

## Introduction

Stress is a widespread issue in society caused by multiple environmental stressors, nowadays underscored by the worldwide pandemic. Children and young people often experience a stress response throughout their education. The impact of stressors on students is at the forefront of the fundamental problems teachers, school psychologists, and other professionals face. Statistics from the United States (American College Health Association [ACHA], 2015) report that four in every 10 university students faced significant stress and that in 32.5% of the cases, the stress response led to decreased academic performance. Excessive stressors in educational institutions cause emotional and physiological health problems, reduce self-worth or hinders healthy self-development (Oku et al., 2015), alter sleep patterns, contribute to poor eating habits, and decrease quality of life (Beiter et al., 2015). Complex demands on academic performance at university, the cumulating effects of exams, and the turbulent and transitional nature of a young adult’s life make students more vulnerable still to various stressors (Karaman et al., 2000). Similarly, stress is a severe problem in younger learners, causing significant academic, social, and health problems (Marques et al., 2015; Wuthrich et al., 2020). Moreover, current pandemic challenges caused a significant increase in stress responses in students worldwide (Ye et al., 2020; Charles et al., 2021) and require provision of preventive programs to alleviate the students’ stress response levels and strengthen their coping resources (Batra et al., 2021; Von Keyserlingk et al., 2021).

Czech research studies also found a severe negative impact of stress on university students (Provazníková et al., 2002). Furthermore, the number of university students with special needs is increasing (Peňáz, 2014). For many of these students, the educational demands result in higher stress response levels and severe health risks. Although university counseling centers offer essential support services, many students would benefit from developing further preventive non-pharmacological strategies, especially those that could offer immediate relief of stress symptoms and help cope with situational demands, thereby contributing to health promotion in educational institutions. Based on results of empirical observations (Skille, 1989), as well as pilot studies (Delmastro et al., 2018; Vilímek et al., 2022), a potential method for coping with acute stress response may be found in vibroacoustic therapy.

**FIGURE 1:**
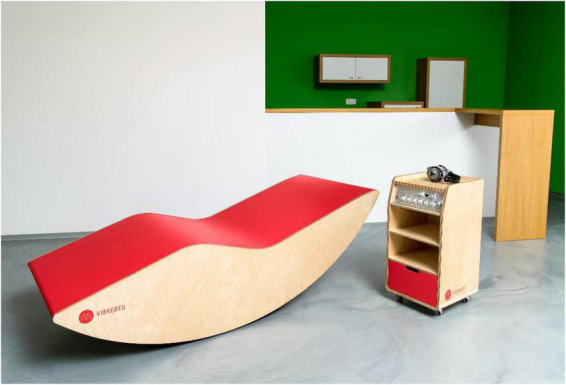
Rehabilitation vibrating bed VIBROBED^®^, participants were supine on it (from the authors’ archive, 2018).

Vibroacoustic therapy (VAT) is defined as “a combination of low frequency sound vibration (and), music listening combined with therapeutic interaction” (Punkanen and Ala-Ruona, 2012, p. 128), although in current vibroacoustic practice the music is not present in all cases (Campbell et al., 2019a). The low frequency sinusoidal sound vibration generally ranges from 20–100 Hz, although this also quite variable. The first prototype for the induction of low frequency sound was created in the second half of the twentieth century by the Norwegian educator Olav Skille and his contemporary in Finland Petri Lehikoinen. Later, due to a sharp increase in modern technology development, new types of vibroacoustic equipment were designed. One modern technology for VAT is a vibracoustic rehabilitation bed called VIBROBED^®^, hereinafter referred to as Vibrobed (see the Figure 1), developed by Vilímek and Švarc from the Czech Republic (2016). Vibrobed consists of:

• A wooden structure,
• Eight vibrating exciters (electrodynamics transducers) distributed according to four body zones (Z1 = calf area, 2×; Z2 = thigh area, 2×; Z3 = cross area, 2×; Z4 = upper back area, 2×),
• A control module (low frequency and auditory music amplifier) and headphones (without LF sound, for auditory music or nature sound only).

By adjusting the type and intensity of vibration, the sound is transmitted to the resonant membrane (special wood composite), and the auditory music (or nature sound) is then played through headphones.

Vibrobed’s innovation lies in modern technological solutions, and in the design of original VAT recordings that make use of the close interaction between low sinusoidal sound and the rhythm of the music. We call this type of low frequency sound (LFS) “sequenced modulation”—the LFS vibration is driven by the rhythmic structure of musical composition, ebbing and flowing with the amplitude of the music, and the parameters of LFS reflect the characteristics of music (see a description of recording called “Elements” in Table 1). The sound recordings developed for Vibrobed are based on the research (Wigram, 1997; Vilímek et al., 2022) of the effect of amplitude modulation of different durations (in seconds), sine sweep up and down in the frequency range 30–80 Hz and various modulations of low-frequency waves in combination with sound and music stimuli.

**TABLE 1:** The vibroacoustic recording called Elements.

Title of composition/ Characteristics	BPM(beats per minute)	Hz–low frequency oscillation	Key of music composition	Description of vibroacoustic composition
Earth	65	30–80 Hz audio generator SWEEP UP 80–30 Hz audio generator SWEEP DOWN–sequential dosing (stimulation/rest)	F# major	Audio generator SWEEP UP-DOWN, panoramic effect (from upper back area to calf area and back), Drum Heart Beat, Instruments—POW-WOW drum, didgeridoo, piano keyboard, soundproof effects.
Fire	106	25–55 Hz audio generator SWEEP UP 45 Hz bass drum	A major	Audio generator SWEEP UP, bass drum, POW-WOW drum, wave drum, percussion, soundproof effects.
Water	75	25–80 Hz electronic bass guitar	A major	Electronic bass guitar—subharmonic generator, wave drum, nature water sounds (river, cave), soundproof effects.
Air	Non-rhythmic structure	33 Hz audio generator–Amplitude modulation	C major	Amplitude modulation 33 Hz—modulated signal, cycle length 16 + 16 s. rest, modulation index 75%. Instruments—piano keyboard, nature water sounds (river, cave), soundproof effects.

Amplitude modulations were first studied by Wigram (1997) and further explored in various experiments by additional researchers (Vilímek et al., 2019, 2022; including authors of this study). Sequenced modulations of LFS make it possible to minimize some secondary complications in frequent vibroacoustic sessions described as overstimulation (Wigram, 1996).

### Effectiveness of vibroacoustic therapy in university students

The effectiveness of VAT has so far been observed mainly through extensive clinical experience. Research has shown the effectiveness of VAT related to persons with different health issues (Wigram, 1996) and some authors (Skille, 1989) also mention the positive effect of VAT on stress response, indicated by the observed effects of VAT on the autonomous nervous system (Delmastro et al., 2018), pulse and blood pressure (Koike et al., 2012), anxiety (Wigram, 1996), or the subjective perception of stress (Ahonen et al., 2013). Based on these findings, VAT may be an easily applied strategy for stress reduction in students with special needs. The advantage of VAT is the immediate relaxation effect, easy and safe usage, and the possibility of home-use and self-application by students themselves. However, the justified extension of VAT as a preventive and therapeutic strategy for stress response regulation needs to be supported by scientific evidence, preferably at the level of randomized controlled trials (RCT) that are so far lacking. It is necessary to systematically determine whether there is any benefit from VAT, a more resource-demanding intervention, compared to music listening only (Linneman et al., 2015), on alleviating or preventing negative stress responses.

We carried out a pilot study to explore the above-mentioned knowledge gap and to develop a protocol for a future randomized control trial. The primary objective of this study is to explore whether applying VAT significantly alleviates the acute stress response in university students. This objective was formulated into the following research question: *“Does the application of low frequency sound with music (recording “Elements”) significantly decrease an acute stress response in university students compared to listening to music without low frequency vibrations?”*

## Methods and analysis

This trial was a parallel, two-armed, superiority randomized controlled trial with 1:1 allocation ratio. Considering it was a pilot study, not all the requirements for randomized control trials, such as allocation concealment or prospective publication of the protocol, were met here (see Strengths and limits of the study). Before the research experiment, all involved persons gave informed consent approved by the Ethics Committee of Faculty of Education, Palacký University in Olomouc, Czech Republic (protocol 5/2019) and all documents were collected by the principal investigator (Jiří Kantor).

### Participants and randomization

The research population consisted of 54 university students of Chinese nationality who participated in summer schools in Olomouc, Czech Republic, organized by the Faculty of Education, Palacký University, in July 2019. Students were included if (a) they wished to voluntarily participate in the study, (b) they were 18–40 years old (most of the students were supposed to be between 20–30 years old), (c) they were of Chinese nationality, and (d) they completed at least 1 week of adaptation following travel to minimize jet-lag. Exclusion criteria were (a) psychiatric diagnosis, (b) neurological disease (e.g., epilepsy, cerebral palsy) or any known contraindication of VAT such as muscle hypotonia, angina pectoris, psychosis, or bleeding (Wigram, 1996), (c) post-traumatic stress disorder, (d) perceived pain, (e) acute sleep deprivation (less than 6 h of sleep), (f) menstruation, (g) stimulant intake (e.g., strong tea, coffee, or smoke) on the day of the experiment or, in the case of stronger, addictive substances (e.g., drugs), 1 day before the experiment.

Two researchers conducted the enrolment through e-mail at the beginning of the summer school and further through personal contact with students. The recruitment process was coordinated and overseen by the recruitment coordinator who created the database of potential participants (Jiří Kantor).

Participants of both sexes were randomized into two sub-samples. The students were allocated into experimental group (VAT) or control group (placebo) based on equal blocked randomization (with block size eight) with stratification according to the sex. The allocation concealment was not conducted. The randomization was implemented by a computerized random number generator (program *Sealed Envelope*).

The intervention was double-blind (to participants and to the statistician). The possibility to influence the results of the experiment during measurements and contact with the participants was reduced due to the manualized data collection/intervention process and the use of objective measurement methods with minimal interaction between the participant and the researcher. A manual available in both Czech and Chinese language was created for the experiment. The manual included standardized instructions for both measurement and intervention personnel to ensure similar conditions for all participants in the experiment. As this trial was intended for healthy young adults, there was no concomitant care and interventions provided, but there was a medical doctor available during and at least 1 h after the experiment in case of any adverse events (pain, vomiting, etc.).

### Research experiment

Prior to the research experiment, all participants in the study had a meeting with one of the researchers to familiarize themselves with the research aims, the course of the experiment, and the inclusion/exclusion criteria. The intervention was realized on two identical Vibrobed vibroacoustic beds. Measurements took place in the natural school environment of the university students, in two similarly equipped rooms at the Faculty of Education, Palacký University in Olomouc. The measurement period was from Monday to Friday in the morning (7:00–11:00) to minimize the effect of circadian oscillation and other factors that affect measurement of autonomous nervous system (ANS) activity (Bilan et al., 2005).

The experimental group intervention consisted of a 20 min of listening to music with low frequency sound. A recording entitled “Elements 2020” was developed originally for this trial (see Table 1). This recording consists of four parts, each lasting 5 min. The control group listened to the same music as experimental group, however, this recording did not contain any low frequency sound in the frequency range 0–100 Hz (it was deleted in the recording studio). The sound volume and vibration intensity was consistently adjusted for each participant according to previous experience (listening starting from low sound intensity), but all participants had the option to optimize the volume and vibration intensity according to their needs by using a control button. In addition, each participant had the possibility (following informed consent) to withdraw from the research experiment at any time, without giving any reasons, but no participant dropped out.

**FIGURE 2:**
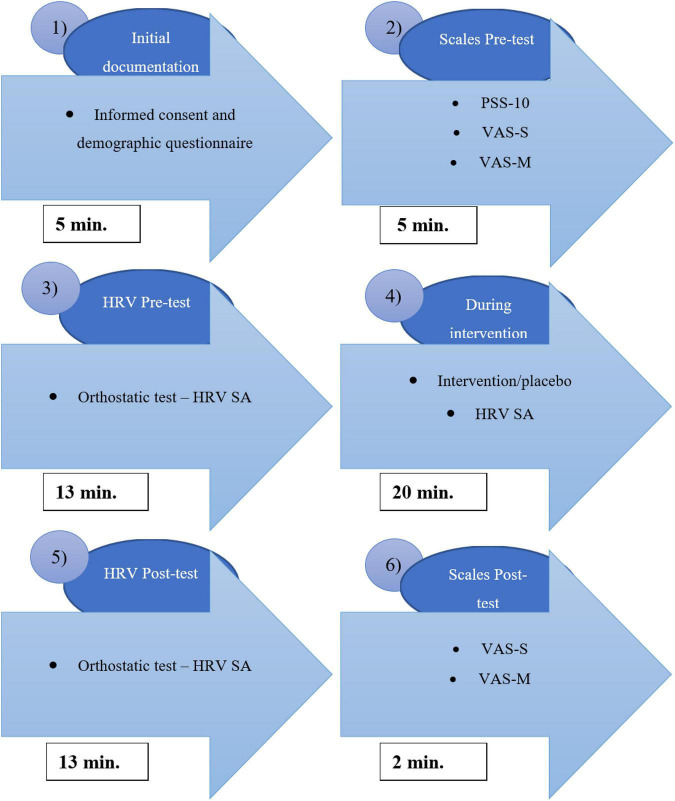
The research experiment procedure (PSS-10, perceived stress scale questionnaire; VAS-S, visual analogue scale for stress; VAS-M, visual analogue scale for muscle relaxation; HRV SA, spectral analysis of heart rate variability).

A diagram of the research experiment can be found in Figure 2 (total time of the experiment for one participant was approximately 50 min).

### The outcome measures and data analysis

Measurements in two parallel groups were carried out before and after intervention by two trained persons under the supervision of a psychologist (Monika Bucharová), a physiological measurements expert (Martin Vítězník), and a research coordinator who supervised adherence to intervention protocols (Jiří Kantor).

This project included measurement of heart rate variability, standardized psychological scales, and a questionnaire developed by the researchers to collect relevant personal/demographic data, as per the suggestion to use a multimodal approach in the assessment of stress (Arza et al., 2018):

Heart rate variability (HRV) enables measuring and quantifying the regulatory effects of the cardiac autonomous nervous system (ANS). Spectral analysis of heart rate variability (HRV SA), one of the methods based on the frequency domain, converts the obtained time data into frequency values with three main components: HF—high frequency (influenced mainly by vagal activity), LF—low frequency (involved in both sympathetic and vagal stimulation), and VLF (very low frequency, probably with the lowest proportion of vagal modulation) (Jakubec et al., 2004). We expected a significant impact on HF. Since there are many factors affecting HRV, we used the orthostatic test that alternates the participant’s standing position (5 min) with the lying position (7 min) to determine the ANS reactivity. Sporttester Polar V800 was used to capture these data in all participants. We checked all HRV SA records for artifacts and manually edited them, if necessary. We used the results as an individual cofactor in interpreting the experiment’s subsequent phases. Subsequently, the interpolated cardiotachogram was calculated, the characteristic parameters of heart rate variability in both the time and frequency domains and the appropriate characteristics of the non-linear analysis were determined. We collected the pre-test and post-test in standing/lying position separately in the experimental and control groups. All physiological data were processed in the Faculty of Biomedical Engineering, Czech Technical University in Prague.

Visual analogue scales for stress (VAS-S)—this single-item assessment of self-reported stress level were administered before and after the intervention to explore any differences in stress perception influenced by LFS. The VAS-S was administered in the form of 10 cm long line, where the extreme left was defined as no stress and the extreme right was defined as maximum perceived stress. VAS-S for stress was selected for this study because of the ease of application while maintaining good psychometric properties (Lesage et al., 2012).

Visual analogue scales for muscle relaxation (VAS-M)—a modification of VAS-M was used to measure a subjective perception of muscle tension/relaxation in the body. The administration was similar—the right extreme defined as no relaxation (max. tension in the body), whereas the left extreme is defined as maximal relaxation (no tension in the body).

Perceived Stress Scale questionnaire (PSS-10) is a widespread, freely available measurement tool with good psychometric properties (Cohen and Williamson, 1988); the Czech version of which was used (Brabcová and Kohout, 2018) to assess whether the level of acute stress before the experiment influences the results of intervention.

A self-constructed questionnaire for personal and demographic data asked respondents about their gender, age, address, general and actual health conditions, medication, menstruation (in women), quality and length of sleep, fatigue (using five-item scale), height and weight, physical activity (in the last 24 h), consumption of alcohol and drugs (in the last 24 h), and food/beverages consumed on the day of measurement. Analysis of the questionnaire’s items enabled a possible statistical analysis of relationships among various factors. These data were important to further control for variables that could significantly affect ANS activity during the experiment. Except in the case of an interfering effect of subjective stress perception (Carrington et al., 2003), which is a dependent variable, anxiety and pain, older age (Almeida-Santos et al., 2016), gender (Koenig and Thayer, 2016), menstrual cycle (Vallejo et al., 2005), sleep quality (Eagles et al., 2016), substance abuse (Ryan and Howes, 2002), food consumption (Nagai et al., 2005), exercise load, and circadian rhythms (Carrington et al., 2003) may also influence the data. Potential interference of these factors was reduced (in this study) by adherence to the inclusion/exclusion criteria.

In order to process the data, Excel-MS, the statistical software R, and the RHRV library (García Martínez et al., 2017) were used. Data were remotely analysed by two specialists (Martin Vítězník, Pavel Smrčka). First, transcribed data were checked for missing values and normal distribution was screened using the Wilcoxon test. Per-protocol analysis was used. The paired samples *t*-test and independent samples *t*-test were used for statistical analysis, with significance level set at *p* < 0.05.

## Results

Twenty-six participants (22 females/four men) were included in the experimental group and 28 (24 females/four men) in the control group. The age range was 18–30 years; most of the participants were 20–22 years, 17 participants were 23–25 years, four participants 26–28 years, and three participants were 29–31 years.

Seven participants did not have breakfast on the day of the experiment and four participants drank a small amount of alcohol the day before the experiment; this was not considered as meeting exclusion criteria. Eleven participants slept at least 8 h, 43 participants 6–7 h, but five participants felt tired (there were no differences between control and experimental groups in sleep and level of fatigue). Eleven participants reported that they had some physical activity a day before the experiment (five subjects from the experimental group and six from the control group).

Data from the Perceived Stress Scale ranged from 13–26 points (mean = 19 points). Most of the participants perceived moderate level of stress, except one participant with low perceived stress level before the experiment. No significant differences were found between the experimental and control groups (Table 2).

**TABLE 2:** *T*-test of PSS-10 in VAT group and PLA group (VAT, vibroacoustic therapy, experimental group; PLA, placebo, control group).

Group	Mean	Standard deviation	*t*	*P*
VAT	18.63	2.86	–0.231	0.818
PLA	18.86	4.44		

### Analysis of physiological data

Heart rate variability SA data from 48 participants were included in the analysis (23 participants from the experimental group and 25 participants from the control group). The reasons for exclusion of six participants were not meeting the eligibility criteria and low quality of data recorded through the Polar V800. The parameters described in Table 3 were chosen for the analysis. These parameters belong to a standard cross-sectional set of parameters in the time, frequency, and non-linear domains. There were significant differences between the groups in pre-test measurements in standing position (LF/HF parameter: *p* = 0.018; ApEn parameter: *p* = 0.008) which indicates a risk of a selective bias. It can be interpreted that the control group was more relaxed in the beginning of the experiment.

**TABLE 3:** Parameters chosen for statistical analysis of HRV SA (spectral analysis of heart rate variability).

Parameter	Area of analysis	Significance
SDNN	Time domain	Standard deviation of NN intervals
pNN50	Time domain	Percentage of successive RR intervals that differ by more than 50 ms
LF/HF	Frequency domain	Ratio of low frequency-to-high frequency power
ApEn	Non-linear domain	Approximate entropy (which measures the regularity and complexity of a time series)
DFA-α1	Non-linear domain	De-trended fluctuation analysis (which describes short-term fluctuations)

Concerning post-test measures, significant differences were found in the lying position in favor of the experimental group for:

• LF/HF parameter: *p* = 0.02, pre-test difference was not significant: *p* = 0.132.
• pNN50 parameter: *p* = 0.028, pre-test difference was not significant: *p* = 0.069.

Moreover, there was a between-groups difference in post-test standing position (parameter ApEn: *p* = 0.016), but at the same time significant pre-test differences were found in the same parameter (*p* = 0.008). Concerning pre-test post-test differences, differences in the SDNN parameter were found for experimental group (*p* = 0.002) as well as control group (*p* = 0.001).

**FIGURE 3:**
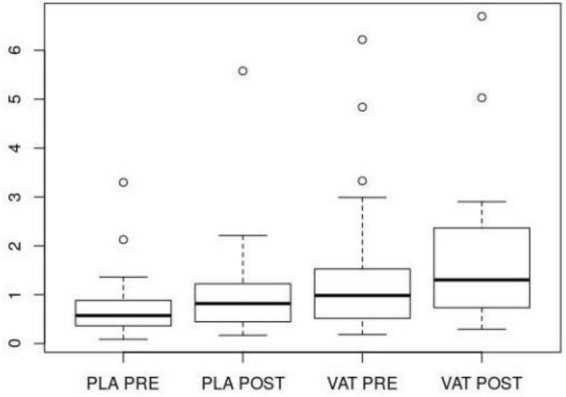
Ratio of low frequency to high frequency power (LF/HF) parameter for lying position (VAT, vibroacoustic therapy, experimental group; PLA, placebo, control group; PRE, pre-test; POST, post-test).

These results (mainly LF/HF parameter, see Figure 3) mean higher activity in the parasympathetic nervous system in the experimental group after the intervention (for lying position) that could be interpreted as a higher level of relaxation after exposure to LFS.

### Analysis of data from visual analogue scales

Visual analogue scales for stress and VAS-M were measured and analysed in all the participants. Forty-four participants in the experiment reported decreased perceived stress after the intervention, five participants perceived increased stress and five participants did not perceive any change in terms of stress. There was a significant pre-test post-test difference for the experimental as well as control group (Table 4), however, no significant difference between groups (Table 5). All participants also completed the VAS-M for which there were also significant differences between pre-test post-test measurements (Table 6), whereas, the between groups difference is not statistically significant (Table 7).

**TABLE 4:** Pre-test post-test difference for VAS-S in experimental (VAT) and control (placebo) group (VAS-S, visual analogue scale for stress; PRE, pre-test; POST, post-test; VAT, vibroacoustic therapy; PLA: placebo).

Test	Mean	Standard deviation	*t*	*P*
PRE-VAT	3.538	2.024	4.723	<0.001
POST-VAT	1.581	1.965		
PRE-PLA	2.929	2.142	4.798	<0.001
POST-PLA	1.154	1.191		

**TABLE 5:** Between groups difference for pre and post-test differences of VAS-S (VAS-S, visual analogue scale for stress; VAT, vibroacoustic therapy; PLA, placebo).

Group	Mean difference	Standard deviation	*t*	*P*
VAT	1.765	1.792	–0.019	0.985
PLA	1.775	1.958		

**TABLE 6:** Pre-test post-test difference for VAS-M in experimental (VAT) and control (placebo) group (VAS-M, visual analogue scale for muscle relaxation; PRE, pre-test; POST, post-test; VAT, vibroacoustic therapy; PLA, placebo).

Test	Mean	Standard deviation	*t*	*P*
PRE-VAT	3.365	1.921	3.973	<0.001
POST-VAT	1.946	2.227		
PRE-PLA	2.704	1.857	2.981	0.006
POST-PLA	1.386	2.104		

**TABLE 7:** Between groups difference for pre and post-test differences of VAS-M (VAS-M, visual analogue scale for muscle relaxation; VAT, vibroacoustic therapy; PLA: placebo).

Group	Mean difference	Standard deviation	*t*	*P*
VAT	1.342	1.838	0.080	0.936
PLA	1.296	2.323		

## Discussion

Results from this pilot trial suggest there is potential for LFS to be used as a means for stress management in an educational environment, e.g., at university. We found that LFS increases parasympathetic nervous system activity and supports the alleviation of subjective stress response and muscle tension. The findings support those from clinical practice, as well as some previous findings (Ahonen et al., 2013; Delmastro et al., 2018; Campbell et al., 2019b; Vilímek et al., 2019, 2021). At the same time, the question remains whether the change was caused mainly by the effect of the auditory music and to what extent LFS may influence physiological and psychological factors connected to stress response. Therefore, in this study the only difference between the experimental and control group was the presence of LFS and we used objective as well as subjective outcome measures to investigate the effect of LFS. The results suggest there may be some difference, but mainly on the physiological level (we detected a significant difference in HRV SA, parameters LF/HF and pNN50). No differences were found between subjective stress perception and relaxation between participants of either group. Our findings do not support those of Veternik et al. (2018) who did not find any significant effect of LFS on the ANS (they used pure sine waves at 20 Hz, 50 Hz, 2 kHz, and 15 kHz). Other studies in the area of vibroacoustic therapy did not explore the effect of LFS on ANS or subjective perception separately from the effect of auditory music. However, the effect of music on ANS is significant (Taylor, 2010) and this also had a significant effect on subjective stress perception and physiological functions in the current study.

This brings us to some methodological problems inherent in the research of vibroacoustic therapy that uses combination of LFS and music and the effect of this two-pronged approach (Chesky et al., 1996; Bartel and Mosabbir, 2021). This could be beneficial in clinical practice but challenging in a research context. Although we tried to separate the effect of music and LFS here, the effect of music used in both groups could mask the subtle changes caused by LFS. This is a problem mainly for exploring the effect of LFS on subjective perception. However, using LFS combined with music listening gave us the possibility of proper blinding, whereas the other options, e.g., comparing LFS to no intervention, would challenge the blinding procedure. If LFS is delivered without music listening, participants easily recognize if an intervention is being offered. There were also discussions about a proper sham for LFS in the vibroacoustic community in recent years as some authors used LFS (Braun Janzen et al., 2019) and others proclaimed the sham was not effective. The problem is that scientific verification is missing that such a sham really does not work and this may challenge the validity of a trial, mainly for researchers outside the vibroacoustic community. This issue does not have an easy solution and in this trial we decided for proper blinding as the effect of placebo on subjective perception may be rather high.

Another problem of combining LFS with another medium of strong effect such as music may be in reduced sensitivity for identification of any physiological or psychological change. Moreover, we did not have the possibility to recruit participants with higher levels of acute perceived stress who would probably have gained greater benefit from the intervention and shown stronger differences pre- and post-stimulus. All of these reasons could influence the strength of the effect of LFS observed in this trial.

Although this pilot trial brings promising results about the effectiveness of LFS, there were also some methodological problems that could have biased the results:

• We did not conceal allocation. Some significant differences in the cardiac vagal activity (measured by HRV) between experimental and control groups before the research experiment were recorded—the differences between sub-samples indicate a risk of selective bias.
• We did not achieve the equal stratification of genders because of lower availability of men in the basic sample.
• Only Chinese students who came for summer school during a short time period from July to August 2019 at the Faculty of Education, University of Palacky in Olomouc were recruited for this trial. We used the homogeneity of this group to our advantage in that they were exposed to similar a situation including demands of adaptation after arrival to foreign country. However, there might be some variables connected to cultural sensitivity of this population that were not controlled here, e.g., the musical background of the participants.
• We did not control for all variables that could have biased the physiological measurements, e.g., exact intake of food, luteal/follicular phase of menstruation cycle, etc. which could have influenced the HRV measurements. On the other hand, we realize that the recruitment process would be much more difficult and would require a long-term study.
• The orthostatic test can reduce the strength of intervention (participants stay in lying position about 7 min before the intervention starts), possibly not being favorable for the outcome measures chosen.

We did not have the possibility to achieve optimal sample size—according to our calculations based on data from this trial, the minimal sample size (for alpha = 0.05; beta = 0.02) is 64 participants [calculation conducted according to Rosner (2011)]. However, we recommend researchers in future studies to aim for an optimal sample size (*N* = 420).

Notwithstanding these limits, the trial has the potential to contribute significantly to current research in VAT because of the low number of research studies, especially studies with physiological measurements. Many authors researching VAT used mainly psychological scales or functional tests such as standing and sitting without pain in minutes (Patrick, 1999; Naghdi et al., 2015). Considering the potentially positive effects of HRV SA on the ANS, there are only a few case studies or small sample case series which are conducted in heterogeneous clinical conditions (Delmastro et al., 2018; Campbell et al., 2019b). Experience from this trial affords the possibility to design a large-scale RCT protocol that is currently registered on clinicaltrials.gov (NCT04293848).

For future research we recommend to:

• Explore different research designs e.g., to use repeated exposure to LFS in every participant and include arms without any intervention. Pooled results from different trails would balance the pros and cons of the study designs.
• Include only participants with higher levels of subjective stress perception. If PSS-10 is used, we recommend to include only participants who score 27 points or more which indicates high perceived stress (Cohen and Williamson, 1988).
• The intervention may have different effects depending on the type of ANS system. Therefore, we also recommend to differentiate the participants according to the sympathotonic and vagotonic type of ANS in future trials.
• Increase the number of participants and ensure equal-gender representation.
• Consider the eligibility criteria and ensure homogeneity of all groups of participants, the same conditions prior to measurement, etc.
• Explore various parameters of LFS, e.g., different frequency or amplitude modulation. In this trial we used different properties of LFS in every composition, but the length of every composition was not sufficient for exploring the differences in the effect on HRV SA.
• We recommend to use both subjective and self-reported scales as well as more objective physiological measurements. Future studies on low frequency vibration and stress response could add other physiological measures, e.g., galvanic skin response or salivary cortisol measurements.

## Conclusion

Vibroacoustic therapy has the potential to contribute to the stress management of university students, as well to the general population with heightened stress response and its general impact on health. This trial proved that LFS has some effect on the parasympathetic nervous system. Since research experiment designs exploring the effect of LFS can be heterogeneous, further studies are needed to understand the potential of LFS on physiological function as well as subjective perception. The experience from this trial helped us to develop a protocol for a large scale RCT and justify future research in this area.

## Data availability statement

The raw data supporting the conclusions of this article will be made available by the authors, without undue reservation.

## Ethics statement

The studies involving human participants were reviewed and approved by Ethics Committee of Faculty of Education, Palacký University in Olomouc. The patients/participants provided their written informed consent to participate in this study.

## Author contributions

ZV, JK, LK, and VR: conceptualization. JK, MV, PS, MJ, VR, and ZV: methodology. JK and VR: recruitment of participants, resources, and funding acquisition. JG, KJ, and GŠ: measurements. MB, PS, and MV: data analysis. JK, MB, EC, MJ, LK, PS, and MV: writing—original draft preparation. MJ, LK, and PS: supervision. JK: project administration. All authors writing—review and editing and approved the submitted version.
